# Late Onset of Chronic Granulomatous Disease Revealed by *Paecilomyces lilacinus* Cutaneous Infection

**DOI:** 10.1007/s10875-021-01140-1

**Published:** 2021-10-01

**Authors:** Clément Lemaigre, Felipe Suarez, Jean-Philippe Martellosio, Cindy Barbarin, Kévin Brunet, Jean Claude Chomel, Ewa Hainaut, Blandine Rammaert, France Roblot, José Miguel Torregrosa-Diaz

**Affiliations:** 1 Service de Maladies Infectieuses Et Tropicales, CHU Poitiers, Poitiers, France; 2 University of Poitiers, Poitiers, France; 3 Necker – Enfants Malades, APHP - Centre Université de Paris, Institut Imagine, INSERM UMR 1163 & CNRS ERL 8254, Université de Paris, Paris, France; 4 Service de Dermatologie, CHU Poitiers, Poitiers, France; 5 INSERM U1070, Poitiers, France; 6 Département Des Agents Infectieux, CHU Poitiers, Poitiers, France; 7 Service d’Oncologie Hématologique Et Thérapie Cellulaire, INSERM CIC 1402, CHU Poitiers, 2 rue de la Milétrie, 86021 Poitiers Cedex, France; 8 Service de Cancérologie Biologique, Poitiers, France

**Keywords:** Chronic granulomatous disease, clonal hematopoiesis, invasive fungal disease, antifungal prophylaxis

## Abstract

Chronic granulomatous disease (CGD) is an inherited immunodeficiency due to defective leukocyte NADPH responsible for recurrent infections and aberrant inflammation. Mutations in the *CYBB* gene are responsible for the X-linked CGD and account for approximately 70% of the cases. CGD is diagnosed during childhood in males. Female carriers may have biased X-inactivation and may present with clinical manifestations depending on the level of residual NADPH oxidase activity. We report the case of a previously asymptomatic female carrier who was diagnosed at age 67 with a skin infection with the rare fungus *Paecilomyces lilacinus* as the first manifestation of CGD. Dihydrorhodamine 123 (DHR) activity was below 10%. Next-generation sequencing (NGS) revealed mutations in *DNMT3A*, *ASXL1*, and *STAG2* suggesting that clonal hematopoiesis could be responsible for a progressive loss of NADPH oxidase activity and the late onset of X-linked CGD in this patient. Long-term follow-up of asymptomatic carrier women seems to be essential after 50 years old.

**Supplementary Information:**

The online version contains supplementary material available at 10.1007/s10875-021-01140-1.

## Introduction

Chronic granulomatous disease (CGD) is a primary immune deficiency associated with reduced NADPH oxidase activity in phagocytes. NADPH oxidase complex is responsible for the production of free radicals leading to oxidative burst and microorganism killing. The different forms of CGD are associated with inactivating mutations in genes coding for the subunits of the phagocyte NADPH oxidase complex. Subunits Gp91, encoded by *CYBB* gene (X-linked), and p22 encoded by the *CYBA* gene (autosomal recessive) are located on the lysosomal membrane. The other subunits encoded by the NCF 1–4 genes (subunits p22, p47, p67, and p40phox) are localized on the cytosolic side of the lysosomal membrane and regulate the catalytic activity of complex [1–3].

The X-linked form is the most common form, usually diagnosed around 5 years old, rarely after 20 [4]. The prognosis is poor due to severe recurrent infections and granulomatous inflammation affecting various organs. Despite antibacterial and antifungal prophylaxis, the mean survival time is 37.8 years in the X-linked form, and 49.6 years in the autosomal recessive form [4]. Although X-linked form preferentially affects men, 8% of cases occur in women due to unfavorable lyonization or new mutation of the active X chromosome [4, 5].

Today, CGD is diagnosed mostly by quantifying the production of hydrogen peroxide (H_2_0_2_) by dihydrorhodamine oxidation (DHR). Susceptibility to infections is apparent in female X-inked CGD carriers with DHR below 20% (and especially below 10%) in particular with opportunistic pathogens such as *Nocardia* spp. and *Burkholderia* spp., and invasive fungi such as *Aspergillus* spp. [1–3]. Autoimmune or inflammatory manifestations of the disease such as discoid lupus erythematosus, Raynaud’s phenomenon, and granulomatous colitis may be present regardless of the DHR level [5].

We report the case of a female carrier who developed a symptomatic form of X-linked CGD with DHR < 10% at the age of 67 years old and hypothesized that the development of clonal hematopoiesis (CH) could be responsible for the late-onset disease.

## Case Description

The patient was a 67-year-old woman, known to be an asymptomatic carrier of X-linked CGD (Gp91 subunit mutation, exon 7, c.676C > T, p.Arg226*) following the death of a daughter due to salmonellosis in 1985. She had two other sons with X-linked CGD, one who died in 1988 at the age of 7 from invasive aspergillosis, and one with a history of recurrent bacterial and fungal infections (aspergillosis) and inflammatory bowel disease, alive and well at age of 35. The patient had never manifested with infections or inflammatory symptoms related to CGD until she was referred to our unit in March 2019. She also had a history of breast cancer treated by tumorectomy, hormonal therapy, and radiotherapy at age 62 years in 2013.

**Fig. 1:**
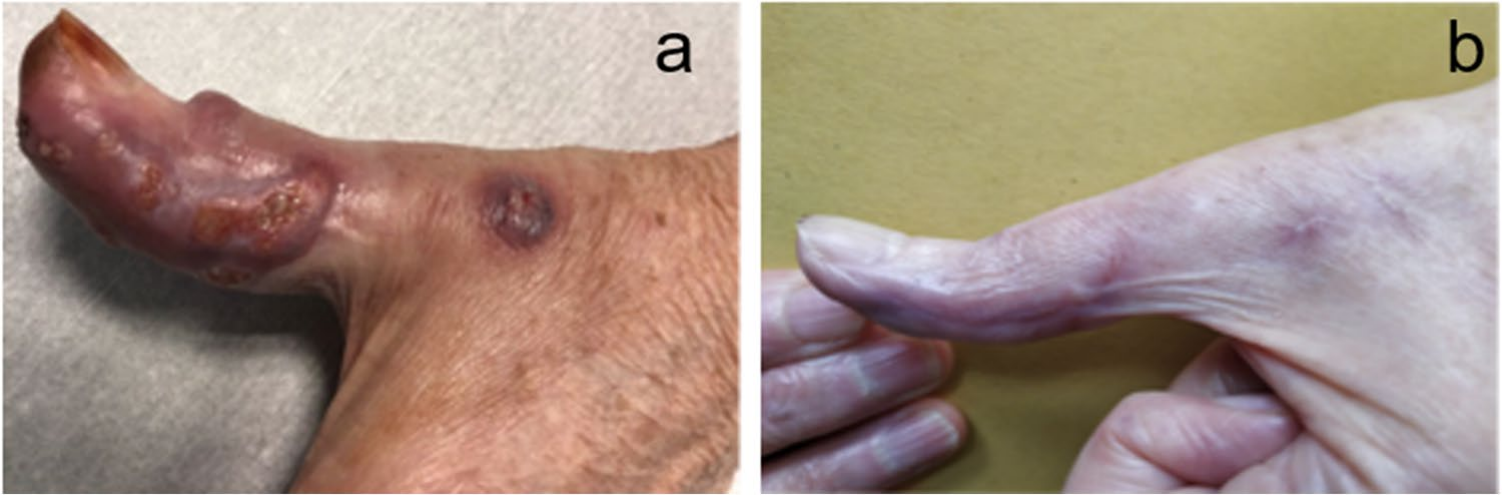
**a** Right thumb before treatment. **b** Right thumb after treatment

The patient presented with skin lesions of her right thumb in December 2018. She had worked as a sheep farmer for many years. The papules evolved towards pustules with purulent discharge on an inflammatory background since December 2018 despite two different lines of oral antibiotic therapy and valaciclovir. Biopsies were performed in March 2019 after she was referred to our department (Fig. 1). All biopsy cultures grew a mold, *Paecilomyces lilacinus*, which was confirmed by sequencing. Histopathological examination showed an inflammatory reaction and mycelial elements compatible with invasive fungal disease. Other microbiological investigations remained negative, including poxvirus PCR. The patient was treated with oral voriconazole, loading dose 400 mg BID then 200 mg BID for 3 months. Treatment was well tolerated and allowed complete healing of the lesions. Her family history and the infection with a rare fungus led us to explore the possibility of immune deficiency linked to CGD. DHR was found to be < 10% consistent with a skewed X-inactivation. No abnormalities in other compartments were detected: Immunophenotypic study of T and NK lymphoid populations revealed a normal quantitative distribution. The B lymphocyte study revealed a discrete B-lymphopenia (5% (84 cells/μL) of CD19 + lymphocytes for a normality interval between 7.2 and 11.2%). There was a normal quantitative distribution of IgA, IgM, and IgG. The complement factors were normal. As the patient had no infectious or inflammatory manifestations of X-linked CGD before age 67, no DHR determination was performed before, but we hypothesized that the severely decreased DHR found was the consequence of an acquired skewing of X chromosome. A routine-implemented next-generation sequencing (NGS) targeted panel was performed in nucleated bone marrow cells. Included genes and exons are available in Supplemental Table 1. The following variants were found: p.Gln573_Ala575del in *DNMT3A* with a variant allele frequency (VAF) of 7.88%, p.Val841Ala in *ASXL1* (VAF 47.94%), and p.Asp651Gly in *STAG2* (VAF 48.26%). The patient had normal blood counts and no signs of myelodysplasia were noted on the bone marrow aspirate, consistent with the presence of clonal hematopoiesis. Prolonged secondary prophylaxis with oral posaconazole and cotrimoxazole was indicated to prevent bacterial and fungal infections in this patient with late-onset severe CGD.

## Discussion

This case addresses the issue of a late-onset CGD manifested by an invasive fungal infection due to a rare environmental mold, *P. lilacinus*, at age 67 in a previously asymptomatic female carrier of X-linked CGD.

Three other cases of late-onset CGD have been described in the literature, all three patients being women aged 45, 53, and 64 [6–8]. These cases were diagnosed after infection by opportunistic pathogens (*Aspergillus* spp. [6], pulmonary infection with *Burkholderia cepacia* [7], and *Serratia marcescens* [8]). Invasive fungal diseases due to molds other than *Aspergillus* spp. are rare during CGD course [9]. *P. lilacinus* is a cosmopolitan saprophytic soil and plant filamentous fungus, with a particular tropism for eyes, skin, and sinuses. The skin is the second most common site of infection after ophthalmic involvement with various unspecific manifestations of the disease from erythematous papules to necrotic lesions or cellulitis [10].

Age-related CH—a frequent phenomenon correlated with medullary aging [11–13]—affects roughly 2–3% of adults over the age of 50 [14]. It is associated with an increased risk of subsequent hematological malignancies and atherosclerosis and could be the key factor to understand the late onset of CGD in our patient. It consists of the emergence of a related cell clone with an acquired gene mutation. The selection of one or more stem cells having a proliferation advantage can gradually replace the other stem cells in some cases [12], leading to reduced genetic diversity of hematopoiesis via the selection of one or more stem cells to the detriment of others. Two major consequences are known to date: increased cardiovascular risk and a variable rate of myelodysplasia and acute leukemia [15, 16].

The impact of CH is however incomplete, as a sort of “incomplete penetrance,” so the influence of some extrinsic factors, as an inflammatory environment, has been proposed. There is an enhanced inflammation seen in patients with CGD, with a mechanism not fully understood but likely related to a defective production of ROS leading to increased expression of NFkB-regulated inflammatory genes [17] and high levels of inflammatory mediators that are expressed in monocytes from patients with X-linked CGD without acute infection compared with controls. So, a variety of inflammatory conditions are seen in CGD patients.

The association of CH and inflammatory/autoimmune disorders has already been reported [18] and there is a growing evidence supporting the association between the XCI skewing and the emergence of autoimmune/non-infectious diseases [19].

Wolach et al. have described for the first time a late onset of X-linked CGD in a 64-year-old woman and have suspected this phenomenon without being able to prove it [8]. In our patient, two of the three mutations most frequently involved in CH (*DNMT3A* and *ASXL1*) [20] were found. DNMT3A gene is located on chromosome 2 and encodes DNA (cytosine-5)-methyltransferase 3 A, an epigenetic modifier essential in hematopoietic stem cell differentiation [21]. It may confer a proliferation advantage becoming more important within stem cell aging [22]. *ASXL1* gene is an epigenetic marker frequently involved in all subtypes of myeloid malignancies and also in clonal hematopoiesis [23].

X-inactivation is an embryological phenomenon that is permanent at each cell division but whose allelic ratio varies throughout life [24]. The reasons of this different XCI with time remain unclear. In this setting, the silencing by long non-coding RNA *Xist* (which variation with time is unknown) and the hematopoietic stem cell senescence remain a valid hypothesis to clarify [25], but their roles have to be determined. As another potential mechanism, the allele-specific loss of methylation with time has been also proposed [25] and could fit best with our hypothesis of the role of clonal hematopoiesis.

Due to the absence of any significant infection until the age of 67 and the depth of the deficit detected in our patient, we hypothesize that it is unlikely that she has had a so deep impaired DHR since birth. The skewed X-inactivation in favor of the mutated *CYBB* gene could have led to the progressive appearance of the genotype and phenotype disease. This asymmetry could be explained by the age-related selection of a cell carrying the mutation [23], as in our patient we found probable CH, so a plausible explanation could be that if the predominant clone carries the *CYBB* mutation, the pattern of inactivation of X becomes asymmetric in favor of this mutation. That would have led to the progressive reduction of the DHR and thus the acquisition of the disease. Interestingly, the patient had been treated by radiotherapy 5 years before, which might have accelerated the acquisition of CH.

Our finding suggests that asymptomatic carriers of *CYBB* mutations should have life-long clinical and biological surveillance. A yearly clinical exam and DHR measurement should start at 50 years old when the risk of clonal hematopoiesis becomes significant, or before if the patient starts to have inflammatory symptoms. Inflammatory symptoms like lupus discoid, pulmonary fibrosis, and Crohn-like disease, can appear before infection when DHR is > 20%. Antifungal and antibacterial prophylaxis should be started as soon as the DHR is < 20%, a percentage below which the risk of infection prevails [5].

In conclusion, the X-linked form of CGD could affect older women due to clonal hematopoiesis. Close monitoring of women carrying the mutation by studying phagocytic function should be interesting to detect DHR decrease below 20%. This strategy could help to prevent infections by starting early chemoprophylaxis and environmental prevention measures.

## Supplementary Information

Below is the link to the electronic supplementary material.

Supplementary file1 (DOCX 19.9 KB)

## Data Availability

Every data used of this work is available upon request.
