# Supplementary material for: Late Onset of Chronic Granulomatous Disease Revealed by Paecilomyces lilacinus Cutaneous Infection
Source: J Clin Immunol. 2021 Oct 1;42(1):60–3. doi: 10.1007/s10875-021-01140-1 (PMC8821496; doi:10.1007/s10875-021-01140-1)
Supplement: Supplementary file 1 — Supplementary file1 (DOCX 19.9 KB) [file 10875_2021_1140_MOESM1_ESM.docx]

## **Supplemental table 1**

| Genes | Analyzed exons | Genes | Analyzed exons | Genes | Analyzed exons | Genes | Analyzed exons |
| --- | --- | --- | --- | --- | --- | --- | --- |
| ***ABL1*** | 4-6 | ***DNMT3A*** | All | ***KDM6A/UTX*** | All | ***RUX1*** | All |
| ***ASXL1*** | 12 | ***ETV6/TEL*** | All | ***KIT*** | 2, 8, 9, 10, 11, 13, 17 | ***SETBP1*** | 4 (partially) |
| ***ATRX*** | 8-10, 17-31 | ***EZH2*** | All | ***KRAS*** | 2,3 | ***SF3B1*** | 13-16 |
| ***BCOR*** | All | ***FBXW7*** | 9-11 | ***MLL*** | 5-8 | ***SMC1A*** | 2, 11, 16, 17 |
| ***BCORL1*** | All | ***FLT3*** | 14, 15, 20 | ***MPL*** | 10 | ***SMC3*** | 10, 13, 19, 23, 25, 28 |
| ***BRAF*** | 15 | ***GATA1*** | 2 | ***MYD88*** | 3-5 | ***SRSF2*** | 1 |
| ***CALR*** | 9 | ***GATA2*** | 2-6 | ***NOTCH1*** | 26, 27, 28, 34 | ***STAG2*** | All |
| ***CBL*** | 8, 9 | ***GNAS*** | 8, 9 | ***NPM1*** | 12 | ***TET2*** | 3-11 |
| ***CBLB*** | 8, 10 | ***HRAS*** | 2, 3 | ***NRAS*** | 2, 3 | ***TP53*** | 2-11 |
| ***CBLC*** | 8, 11 | ***IDH1*** | 4 | ***PDGFRA*** | 12, 14, 18 | ***U2AF1*** | 2, 6 |
| ***CDKN2A*** | All | ***IDH2*** | 4 | ***PHF6*** | All | ***WT1*** | 7, 9 |
| ***CEBPA*** | All | ***IKZF1*** | All | ***PTEN*** | 5, 7 | ***ZRSR2*** | All |
| ***CSF3R*** | 14-17 | ***JAK2*** | 12-14 | ***PTPN11*** | 3, 13 |  |  |
| ***CUX1*** | all | ***JAK3*** | 13 | ***RAD21*** | All |  |  |
